# Breathing Exercise Called the Maximal Abdominal Contraction Maneuver

**DOI:** 10.3390/medicina57020129

**Published:** 2021-02-02

**Authors:** Jung Won Kwon, Seo Yoon Park, Ki Hyun Baek, Kyoungsoo Youk, Seunghue Oh

**Affiliations:** 1 Department of Physical Therapy, College of Health Sciences, Dankook University, 119, Dandae-ro, Dongnam-gu, Cheonan-si, Chungnam 330-714, Korea; kjwonpt@hanmail.net; 2 Department of Health, Graduate School, Dankook University, 119, Dandae-ro, Dongnam-gu, Cheonan-si, Chungnam 330-714, Korea; pgy0614@hanmail.net (S.Y.P.); backho86@naver.com (K.H.B.); 3 Department of Health Welfare, College of Health Sciences, Dankook University, 119, Dandae-ro, Dongnam-gu, Cheonan-si, Chungnam 330-714, Korea; 3396599@naver.com

**Keywords:** breathing exercise, abdominal muscle, core muscle, co-contraction, ultrasound images

## Abstract

*Background and objectives:* The maximal abdominal contraction maneuver (MACM) was designed as an effective and efficient breathing exercise to increase the stability of the spinal joint. However, it has not been determined whether MACM is more effective and efficient than the maximal expiration method. Thus, the present study was undertaken to investigate whole abdominal muscle thickness changes after MACM. *Materials and Methods:* Thirty healthy subjects (17 males and 13 females) participated in this study. An experimental comparison between MACM and the maximal expiration task was conducted by measuring the change of abdominal muscle thickness such as the transverse abdominis (TrA), internal oblique (IO), external oblique (EO) and rectus abdominis (RA) using ultrasound images. *Results:* The results indicated that MACM resulted in significantly greater muscle thickness increases of the TrA and RA than the maximal expiration exercise (*p* < 0.05). *Conclusion:* MACM provided better exercise than the maximal expiration exercise in terms of increasing spine stability, at least from a co-contraction perspective.

## 1. Introduction

The abdominal muscles pull the abdominal wall inward and increase intra-abdominal pressure [1,2]. In particular, abdominals can also be regarded as powerful expiration muscles [3], and are well-known vital components of the core muscle [4,5,6]. Core muscle recruitment enhances core stability and helps provide proximal stability to facilitate distal mobility [3,7]. In addition, core muscles must contract in sequence with appropriate timing and tension to achieve optimal core stability [7]. Therefore, abdominal muscles are thought to contribute to core stability as well as being respiratory muscles.

Abdominal muscle activation can increase spinal joint stability [3,8], and failure of these muscles to contract sufficiently may lead to spinal joint instability [9]. The most popular method used to address spinal instability is the abdominal drawing-in maneuver (ADIM), which is commonly used in lumbar stabilization training programs [8,10]. ADIM effectively activates the transverse abdominis (TrA) [11], and several studies have shown the TrA muscle training can increase spinal joint stability [12,13,14]. However, it is difficult and takes much time to train patients how to perform ADIM, and some attempts have been made to overcome these difficulties, such as maximal expiration [9]. Recent studies have shown that maximal expiration provides a useful means of inducing co-contraction of deep and superficial abdominals [5,9], and co-contraction of these abdominal muscles appears to have greater benefits than ADIM in terms of improving lumbar stabilization because all abdominal muscles contribute to core stability [9]. In addition, it has also suggested a combination of maximal expiration and exercise might cause greater abdominal muscle activation [3].

Recently, our research team has designed a breathing exercise maneuver called the maximal abdominal contraction maneuver (MACM) as an effective and efficient breathing exercise that increases spinal joint stability. MACM focuses on maximal co-contraction of all abdominal muscles and induces more muscle contraction than maximal expiration. Furthermore, MACM includes lower hip adductor muscle contraction and maximal expiration. However, it has not been previously investigated how MACM compares with the maximal expiration method in terms of effectiveness and efficiency.

Therefore, we investigate changes in whole abdominal muscle thickness after MACM and the maximal expiration method.

## 2. Method

### 2.1. Participants

The study subjects were 30 healthy adults (17 men and 13 women). The general characteristics of the subjects are shown in Table 1. The study inclusion criteria were as follows: (1) no respiratory disease or taking of any related drug; (2) no lesion that may have affected the experiment; (3) the ability to understand and follow the experiment; (4) no capable of interfering with ultrasonography image analysis; (5) no hip or spinal orthopedic operation or surgery, and the ability to fully adduct hips; (6) no history of rib fracture; and (7) the ability to breathe without pain. All participants were given full instructions and agreed to participate in the study. The experiment was conducted with the Institutional Review Board (code: IRB, 2019-11-016-002; date: 30 December 2019) approval of Dankook university.

### 2.2. Apparatus

Ultrasound images were obtained using a SONOACE R7 ultrasonograph (Medison, Samsung, Seoul, Korea). An 8 MHz linear transducer was used to measure thicknesses of abdominal muscles. The transducer was oriented transversely, perpendicular to the abdominal musculature to align with the fibers of abdominal muscles [15,16]. The target muscles were the rectus abdominis (RA), external oblique (EO), internal oblique (IO), and transverse abdominis (TrA). To measure EO, IO, and TrA thicknesses, the linear transducer was placed along the lateral abdominal wall on the midaxillary line superior to the iliac crest. To measure RA thicknesses, the linear transducer was placed at 2.5 cm lateral to the umbilicus. Note that studies using ultrasound images can quantify changes in muscle thickness, and it is well known that ultrasound is a useful tool to assess muscle function [17]. Most studies investigating the breathing exercise and abdominal muscle using ultrasound images have taken images at the end of expiration [18,19]. Additionally, a previous study reported that the abdominal muscle thickness increased according to lung volume decreased due to expiration [20]. The current study focused on the maximal thickness of the abdominal muscles (RA, EO, IO and TrA) according to breathing exercise. Thus, the image capturing was performed at the end of expiration.

### 2.3. Protocol

**Figure 1:**
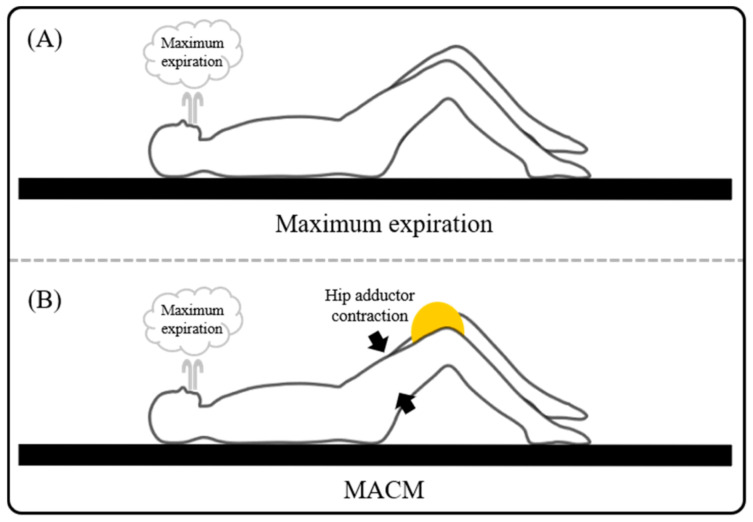
The breathing exercise of maximum expiration and maximal abdominal contraction maneuver (MACM). (**A**) Maximum expiration. (**B**) MACM.

**Figure 2:**
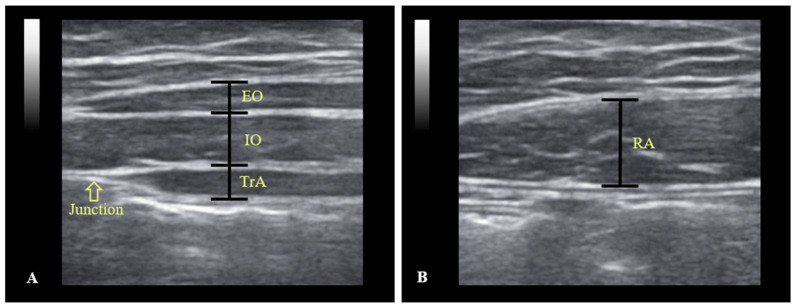
Example ultrasound images of the abdominal muscles. (**A**) Ultrasound image of external oblique (EO), internal oblique (IO) and transversus abdominis (TrA). (**B**) Ultrasound image of rectus abdominis (RA).

The following two tasks were performed in this study, that is, (1) MACM (Figure 1B) and (2) only maximal expiration (Figure 1A) in a hook lying position. Note that in this position the hip joints were at 45 degrees of flexion and the knee joints were at 90 degrees of flexion in the supine position. Before the experiment, subjects were instructed how to perform each task during a 10 min practice session. Subjects performed the MACM and maximal expiration task in randomized order and each task was performed four times (twice to measure RA thickness, twice to measure EO, IO, and TrA thicknesses). The verbal instruction for the maximal expiration task was, “Breathe out maximally and hold your breath”, and for MACM task was, “Squeeze a ball located between knees during maximal expiration”. As subjects approached the end of expiration, an operator captured ultrasound images. Additionally, the baseline images also captured at the resting phase. Image capturing was repeated twice for each abdominal muscle such as RA, EO, IO and TrA. A 30 s rest was provided between repetitions of the exercise. A 3 min rest was provided between each of the exercise to prevent muscle fatigue.

### 2.4. Data Analysis

The thickness of change of each abdominal muscle was measured twice and the average values were calculated. Here, the change of muscle thickness was calculated by subtracting in the resting phase from average maximal thickness (in end of expiration). The thickness of each muscle was determined using on-screen calipers. The thicknesses of the EO, IO, and TrA were measured by drawing a perpendicular line at 1 cm from the edge of the aponeurosis of abdominal muscles (Figure 2A). RA thickness was defined as the longest length in a perpendicular line of the muscle from the center on the ultrasound image (Figure 2B). The paired t-test in SPSS Ver. 22.0 (SPSS Inc., Chicago, IL, USA), was used to determine the significances of task-related muscle thickness differences. Statistical significance was accepted for *p*-values <0.05.

## 3. Results

**Figure 3:**
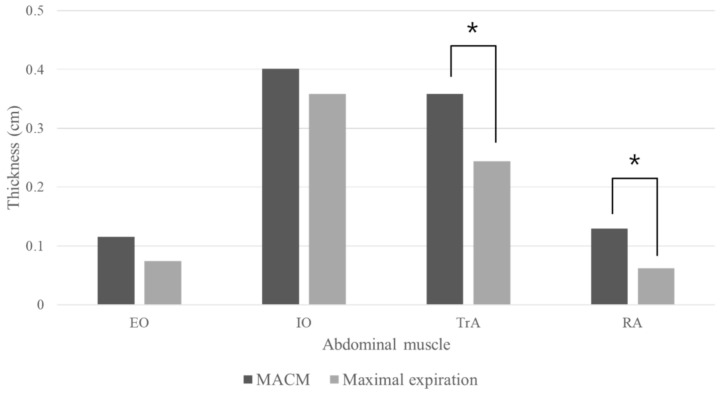
Result of mean muscle thickness. EO: external oblique muscle; IO: internal oblique muscle; TrA: transverse abdominis; RA: rectus abdominal muscle; MACM: maximal abdominal contraction maneuver. * There was a significant difference between the MACM and maximal expiration (*p* < 0.05).

Muscle thicknesses after MACM and maximal expiration tasks are provided in Figure 3. MACM increased muscle thicknesses of the RA and TrA significantly more than maximal expiration (*p* < 0.05), but increased EO and IO muscle thickness non-significantly more than maximal expiration (*p* > 0.05).

## 4. Discussion

Recent studies have indicated expiration during co-contraction of abdominal muscles may be beneficial because it increases the activations of abdominal muscles [3,21]. Additionally, abdominal muscle contraction has been shown to increase spine stiffness and vertebral segment stability [22,23]. Based on these observations, our research team designed a breathing exercise method called MACM that involves the contraction of all abdominal muscles. To validate the MACM breathing exercise, we compared changes in abdominal muscle thicknesses after MACM and the maximal expiration exercise.

We found that MACM increased the thicknesses of EO, IO, TrA, and RA muscles more than maximal expiration, though differences were only significant for TrA and RA. These increases of muscle thickness are probably caused by hip adduction accompanied by abdominal muscle co-contraction. Hip adductors originate proximal to the inferior aspect of the body and ischium, insert distally on the femur and influence the control of trunk muscles attached to the pelvis [24]. These muscles contraction plays a role in the contraction of abdominal and pelvic floor muscles, and contribute to spinal stability [24,25,26]. Thus, hip adductor contraction during MACM might synergistically facilitate increased abdominal muscle activity that reinforces trunk muscles and contributes to spinal stability [24]. Several studies have reported lower limb movement enhances abdominal muscle co-contraction [3,24,25,27]. In 1983, Hemborg et al. showed that bridge exercise with a ball between the knees improved core stability by inducing co-activation of trunk and pelvic floor muscles such as the TrA and RA [28]. These results are consistent with our result that MACM with additional lower limb movement can induce more contraction of TrA and RA muscles than maximal expiration.

At least from a theoretical perspective, MACM appears to be inappropriate for retraining abdominal muscles in patients with lower back pain (LBP), because abdominal muscles must contract in sequence with appropriate timing and tension to achieve optimal core stability [7]. Recent reports suggest individual abdominal muscles differently contribute to respiration [29,30], which suggests MACM may not be ideal for achieving contraction of specific abdominal muscles during the early phase of motor rehabilitation. However, forceful contraction of abdominal muscles is necessary to generate intra-abdominal pressure, which is known to reduce spine compression force [26,31]. Thus, it seems co-contraction of all abdominal muscles by MACM increases intra-abdominal pressure, and that forceful co-contraction of abdominal muscles by MACM might provide some load relief of spine compression force [26]. Additionally, in 2007, Grenier et al. reported that the bracing effect created by the all abdominal muscle co-contraction provides greater lumbar spine stability than specific transversus abdominis recruitment [32], and thus bracing effect created patterns that better enhance stability [32,33]. Therefore, MACM offers an effective co-contraction training method based on providing intra-abdominal pressure and lumbar spine stability rather than maximal expiration.

Several studies have reported that maximal expiration might usefully increase muscle co-contraction [3,5,9]. In 2005, Hodges et al. showed that greater breathing effort leads to greater abdominal and lower back muscle activities and increases spinal stiffness [34]. Additionally, in 2015, Ishida et al. demonstrated that side bridge exercise combined with maximal expiration enhanced abdominal muscle activities [3]. These studies suggest abdominal muscle co-contraction with additional movement increases vertebral segment stability. For this reason, we designed the MACM exercise which focuses on the co-contraction of abdominal muscles.

The present study confirms that the MACM can result in more abdominal muscle co-contraction than maximal expiration, which suggests MACM offers a means of overcoming the limitations of ADIM and that MACM is more effective than maximal expiration in terms of contracting the TrA and RA muscles. However, the study has its limitations. In particular, it should be noted the study was conducted on healthy adults. Additional studies are also required on the persistence of the effects of MACM using various clinical tools. Moreover, there are limitations to generalizing the current results to those who have difficulty analyzing ultrasound images.

## 5. Conclusions

By comparing muscle thickness increases, we found that MACM causes significantly more muscle co-contraction than maximal expiration. This result suggests that MACM exercise provides a better means of increasing co-contraction and spine stability than maximal expiration.

## Figures and Tables

**Table 1:** General characteristics of subjects.

	Number of Subjects	Age	Height (cm)	Weight (kg)	Body Mass Index (kg/m^2^)
Male	17	23.71 (1.65)	173.18 (6.10)	72.71 (12.89)	24.17 (3.54)
Female	13	22.15 (1.46)	162.08 (5.42)	56.45 (7.87)	21.49 (2.91)

Values represent mean (± standard deviation).

## Data Availability

The data presented in this study are available on reasonable request from the corresponding author.
